# Characteristic Sounds Facilitate Object Search in Real-Life Scenes

**DOI:** 10.3389/fpsyg.2019.02511

**Published:** 2019-11-05

**Authors:** Daria Kvasova, Laia Garcia-Vernet, Salvador Soto-Faraco

**Affiliations:** ^1^Center for Brain and Cognition, Universitat Pompeu Fabra, Barcelona, Spain; ^2^ICREA – Catalan Institution for Research and Advanced Studies, Barcelona, Spain

**Keywords:** visual search, attention, semantics, natural scenes, multisensory, real life

## Abstract

Real-world events do not only provide temporally and spatially correlated information across the senses, but also semantic correspondences about object identity. Prior research has shown that object sounds can enhance detection, identification, and search performance of semantically consistent visual targets. However, these effects are always demonstrated in simple and stereotyped displays that lack ecological validity. In order to address identity-based cross-modal relationships in real-world scenarios, we designed a visual search task using complex, dynamic scenes. Participants searched for objects in video clips recorded from real-life scenes. Auditory cues, embedded in the background sounds, could be target-consistent, distracter-consistent, neutral, or just absent. We found that, in these naturalistic scenes, characteristic sounds improve visual search for task-relevant objects but fail to increase the salience of irrelevant distracters. Our findings generalize previous results on object-based cross-modal interactions with simple stimuli and shed light upon how audio–visual semantically congruent relationships play out in real-life contexts.

## Introduction

Interactions between sensory modalities are at the core of human perception and behavior. For instance, the distribution of attention in space is guided by information from different sensory modalities as shown by cross-modal and multisensory cueing studies (e.g., Spence and Driver, 2004). Most research on cross-modal interactions in attention orienting has typically employed the manipulation of spatial (Spence and Driver, 1994; Driver and Spence, 1998; McDonald et al., 2000) and temporal (Busse et al., 2005; Van der Burg et al., 2008; van den Brink et al., 2014; Maddox et al., 2015) congruence between stimuli across modalities. However, recent studies have highlighted that in real-world scenarios, multisensory inputs do not only convey temporal and spatial congruence but also bear semantic relationships. The findings of these studies have shown that cross-modal correspondences at the semantic level can affect detection and recognition performance in a variety of tasks, including the distribution of spatial attention (e.g., Molholm et al., 2004; Iordanescu et al., 2008, 2010; Chen and Spence, 2011; Pesquita et al., 2013; List et al., 2014). For instance, in visual search among images of everyday life objects, sounds that are semantically consistent (albeit spatially uninformative) with the target speed up search times, in comparison to inconsistent or neutral sounds (Iordanescu et al., 2008, 2010). However, one paramount question which remains to be answered in this field is, to which extent such multisensory interactions discovered under simplified, laboratory conditions, have an impact under the complexity of realistic, multisensory scenarios (Matusz et al., 2019; Soto-Faraco et al., 2019). We set out to address this question.

**FIGURE 1:**
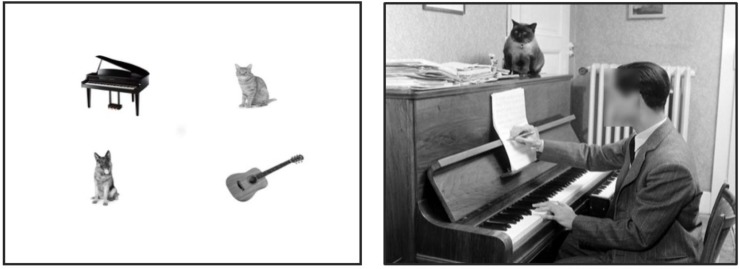
Left picture is an example of stimuli used as a typical search array in a search experiment. Figures are randomly chosen and randomly distributed in space without any meaningful connection between them. On the right naturalistic picture some objects are the same as on the left but now they are put into a context with spatial envelope, proportionality, and variety of meaningful and functional connections between objects.

Previous findings on cross-modal semantic effects on search behavior so far have used static, stereotyped artificial scenarios that lack meaningful context (Iordanescu et al., 2008, 2010; List et al., 2014). However, searching targets in these simplified displays used in laboratory tasks is very different from the act of looking for an object in complex, naturalistic scenes. As many authors have pointed out before, the generalization of laboratory findings using idealized materials and tasks is often far from trivial (Matusz et al., 2019, for a recent review). Outcomes that are solid and replicable under these simplified conditions may turn out differently in contexts that are more representative of real life (Wolfe et al., 2005; Maguire, 2012; Peelen and Kastner, 2014, for examples in visual research; see Soto-Faraco et al., 2019, for a review concerning multisensory research). First, realistic scenes are usually far more cluttered than stereotyped search arrays. Second, natural scenarios provide organization based on relevant prior experience: When searching for your cat in the living room, you would not expect the cat hovering midway to the ceiling, next to a floating grand piano. Yet, many laboratory tasks require just that: A picture of a (target) cat can be presented within a set of randomly chosen objects that have no relations between them, arranged in a circle, against a solid white background (Figure 1).

Previous visual-only studies have already made a point about the differences in how spatial attention is distributed in naturalistic, real-life scenes compared to simple artificial search displays typically used in psychophysical studies (e.g., Peelen and Kastner, 2014, for a review; Henderson and Hayes, 2017). Given that experience and repetition tends to facilitate visual search (Shiffrin and Schneider, 1977; Evans et al., 2013; Kuai et al., 2013), another important difference could lie in our familiarity (and hence, predictability) with natural scenes, compared to laboratory displays. In addition, humans can extract abundant information from natural scenes (gist) at a glance, quickly building up expectations about the spatial layout and relationships between objects (Biederman et al., 1982; Greene and Oliva, 2009; Peelen et al., 2009; MacEvoy and Epstein, 2011).

For example, Nardo et al. (2014) reported that cross-modal semantic congruency between visual events and sounds had no effect on spatial orienting or brain activity during free viewing of videos from everyday life scenes. In contrast, another study by Mastroberardino et al. (2015) with static images reported that visual images could capture spatial attention when a semantically congruent, albeit spatially uninformative sound was presented concurrently. Along with a similar line, Iordanescu et al. (2008, 2010) showed that spatially uninformative characteristic sounds speeded up the visual search when consistent with the visual target. Conversely to the study of Nardo et al. (2014), which found no effect, Iordanescu et al. (2008, 2010) and Mastroberardino et al. (2015) used simple static images presented in decontextualized search arrays (Iordanescu et al., 2008, 2010). Both, these differential features (dynamic nature of natural scenes and their complexity) have been pointed out as important components for the generalization of cognitive psychology and neuroimaging findings to real-world contexts (e.g., Hasson et al., 2010). Another possible important variable in prior research on cross-modal semantic influence on attention is task-relevance. Unlike Nardo et al. (2014) and Mastroberardino et al. (2015) studies, in the study of Iordanescu et al. (2008, 2010) the critical (target) objects were task-relevant, potentially making audio–visual congruence relations also relevant to the task.

Based on the results of these prior studies, one first outstanding question is whether cross-modal semantic relationships can play a role at all in complex dynamic scenarios. Until now, the only study using such scenarios (Nardo et al., 2014) has returned negative results, in contrast with other studies using more stereotypical displays (Iordanescu et al., 2008, 2010; Mastroberardino et al., 2015). Given that a major difference between these studies was task relevance of the cross-modal events, a second interrelated question is whether the impact of cross-modal semantic relationships, if any, is limited to behaviorally relevant events. Here we present a study using a novel search task on realistic scenes, in order to shed light on these two questions.

In our visual search protocol, targets were everyday life objects appearing in video clips of naturalistic scenes. Spatially uninformative characteristic sounds of objects mixed with ambient noise were presented during search. The relationship between the object sounds and the visual target defined four different conditions: *target-consistent sound*, *distracter-consistent sound*, *neutral sound*, and *no sound*, which was a baseline condition that contained only background ambient noises. Visual search performance was measured with reaction times.

We hypothesized that, if cross-modal semantic congruency guides attention in complex, dynamic scenes, then reaction times should be faster in the target-consistent condition than in the distracter-consistent, neutral, or no sound conditions (e.g., target-consistent characteristic sounds will help attract attention to the corresponding visual object). Regarding the possible task-relevance modulation of cross-modal semantic effects, we hypothesized that if audio–visual semantic congruence attracts attention in natural scenes automatically even when the objects are irrelevant to the current behavioral goal, then one should expect a slowdown in responses to targets in distracter-consistent trials, with respect to neutral sound trials. Else, if audio–visual semantic congruence has an impact only when task-relevant (as we expected), then distractor-congruent sounds should not slow down performance compared to other unrelated sounds. In order to check the potential unspecific effects of object sounds on visual search times, such as alerting (Nickerson, 1973), we included neutral sound condition as a control. Neutral sounds were sounds that did not correspond to any object in the video of the current trial. Thus, we expected that differences due to general alerting of sounds, if any, would equally affect target-consistent, distractor-consistent, and neutral sound conditions, but not the no-sound baseline.

## Materials and Methods

### Participants

Thirty-eight volunteers (12 males; mean age 25.22 years, *SD* = 3.97) took part in the study. They had normal or corrected-to-normal vision, reported normal hearing, and were naïve about the purpose of the experiment. All subjects gave written informed consent to participate in the experiment. Two subject-wise exclusion criteria were applied before any data analysis. (1) If the false alarm rate in catch trials (trials in which the search target was not present) was above 15%. (2) If accuracy in one or more conditions was <70%. After applying these criteria, we retained data from 32 participants.

### Stimuli

#### Visual Stimuli

A set of 168 different video-clips were obtained from movies, TV shows, and advertisement, and others were recorded by experimenters from everyday life scenes. The video clips, size 1024 × 768 pixels, and 30 fps were edited with Camtasia 9 software^1^ to 2 s duration fragments. No fades were used during the presentation. Ninety-six videos were used for the experimental conditions described below, and 72 videos for catch trials. For all of the videos, the original soundtrack was replaced with background noise created by the superposition of various everyday life sounds (see example video clips and sounds in the Supplementary Materials).

Each video clip used for experimental (target-present) conditions contained two possible visual targets, which were always visual objects which have a characteristic sound (such as musical instruments, animals, tools, etc.). The criteria to choose the target objects in the videos was that, although they were visible (no occlusions, good contrast), they were not part of the main action in the scene. For instance, if a person is playing guitar and this is the main action of the scene, the guitar could not be a target object. However, in a scenario with a band playing different instruments, the guitar could be a possible target. Both target and distractor objects are presented from the beginning till the end of the video except for the catch trials where neither target or distractor are presented. We applied this criterion to make the search non-trivial. Nevertheless, in order to compensate for potential biases related to particular objects or videos, we counterbalanced the materials so that each video and object contributed as a target and as a distractor in equal proportions across participants (see the section “Procedure”).

#### Auditory Stimuli

We used characteristic sounds that corresponded semantically to the target/distractor objects (e.g., barking dog). However, they gave no information about the location of the object (sounds were always central) or its temporal profile (the sound temporal profile did not correlate with visual object motion or appearance). All the sounds were normalized to 89 dB SPL and had a duration of 600 ms. Sounds were delivered through two loudspeakers placed at each side of the monitor, in order to render them perceptually central.

### Procedure

The experiment was programmed and conducted using the Psychopy package 1.84.2 (Python 2.7) running under Windows 7. Participants were sitting in front of a computer monitor 22.5″ (Sony GDM-FW900) at a distance of 77 cm. We calibrated the video and sound onset latencies using The Black Box Toolkit^2^ (United Kingdom), within an error of *SD* = 7.34 ms.

In order to start each block of the experiment, participants pressed the space bar. Each trial started with a cue word printed on the screen indicating the target of the visual search for that trial. After 2000 ms, a video clip with the background noise plus, if applicable, a characteristic object sound of the corresponding condition (target-consistent, distracter-consistent, neutral) were presented. Following previous laboratory studies that used complex sounds and visual events we decided to desynchronize presentation of the audio–visual event, by presenting the sound 100 ms before the video onset (Vatakis and Spence, 2010, for review; Knoeferle et al., 2016, for a similar procedure).

**FIGURE 2:**
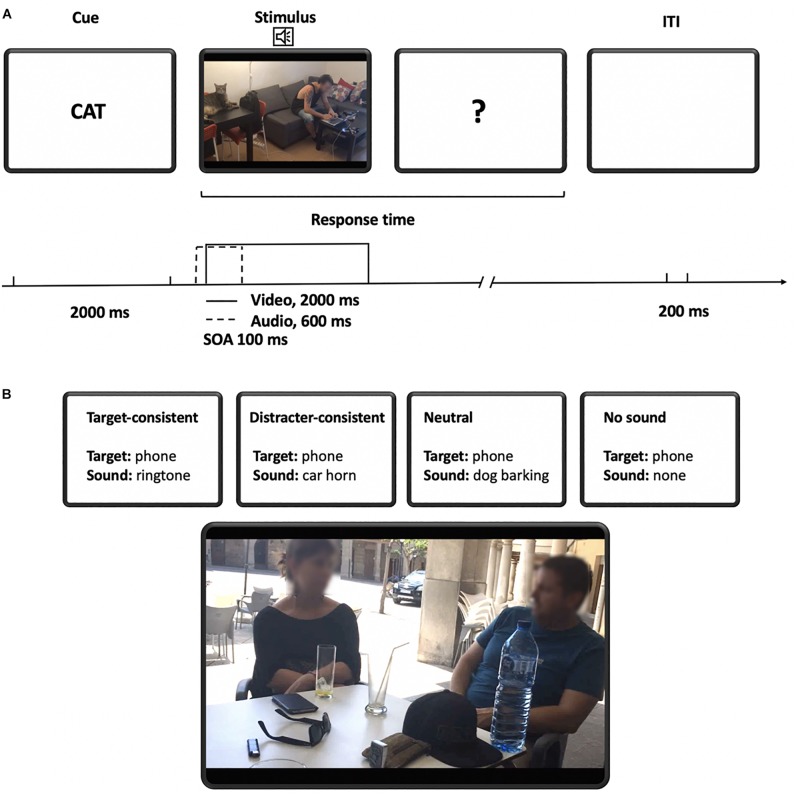
**(A)** Sequence of events in the experiment. The trial started with the presentation of target word for 2000 ms. The target word was followed by the auditory cue and video. Auditory cue was presented 100 ms before the video was shown (SOA = 100 ms) and lasted for 600 ms while the video lasted for 2000 ms. There was no time limit for the participant response. 200 ms after the participant had responded a new target word was presented. **(B)** Example of conditions. In this example of stimulus, the possible targets are a mobile phone and a car. If the target is a mobile phone, in the target-consistent condition the sound will match the target, in the distractor-consistent condition the sound will match the distractor (a car), in the neutral condition the sound will not match any object of the scene (e.g., dog barking), and in the no sound condition there will be just background noise and no auditory cue. The image is a frame of the video clip filmed by the research group.

The participant’s task was to judge whether or not the pre-specified target object was present in the video clip as fast as possible and regardless of its location. If the video ended before participants’ response, a question mark showed up on the screen and stayed there until the participant responded. The next trial started 200 ms after the participant had responded (Figure 2). Half of the participants had to press A key (QWERTY keyboard) as soon as they found the target object. In case the object was not present on the scene, they pressed L key. For the other half it was the other way around. Visual search performance for each subject and condition was determined by the mean response time (RT) of correct responses.

**FIGURE 3:**
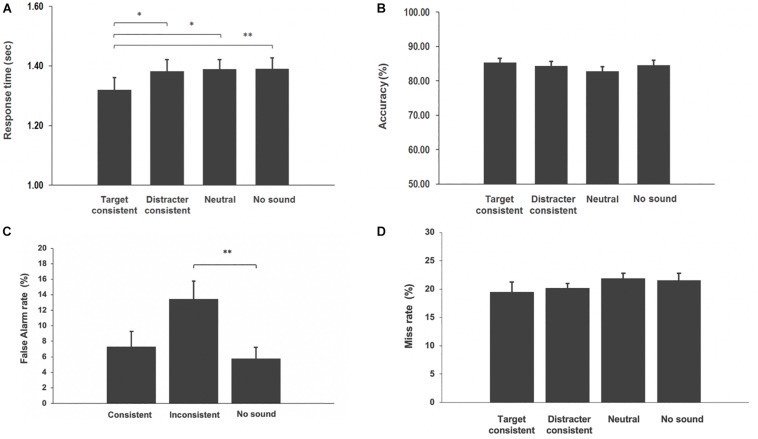
**(A)** Visual search reaction times toward a target and error rates were plotted in the target consistent sounds, distracter-consistent sounds, neutral sounds, and no sound conditions. Error bars indicate the standard error. Asterisks indicate significant difference between conditions (^∗^*p*-value < 0.05, ^∗∗^*p*-value < 0.01). **(B)** Visual search accuracy toward a target and error rates were plotted in the target-consistent sounds, distracter-consistent sounds, neutral sounds, and no sound conditions. Error bars indicate the standard error. **(C)** False alarm rates were plotted in the conditions when sound was consistent with the cue, inconsistent with the cue, and in the no sound condition. **(D)** Miss rates were plotted in the target-consistent sounds, distracter-consistent sounds, neutral sounds, and no sound conditions. Error bars indicate the standard error.

Four types of sound–target conditions were used: *target-consistent*, *distractor-consistent*, *neutral*, and *no sound*. In the target-consistent condition, the identity of the sound matched with the target object. In the distractor-consistent condition, the sound matched a non-target (distracter) object present in the scene. In the neutral condition, the object sound did not match any of the objects in the scene. Finally, in the baseline condition, no particular object sound (an auditory cue) was present (besides the background noise) (Figure 3).

Due to the high heterogeneity of the video-clips, we decided to counterbalance them across conditions and participants. Each participant saw each video-clip once, but overall, each video clip appeared in each of the four experimental conditions the same number of times (across subjects), except for trials which were the same for all participants. To achieve this, we created a total of eight different versions of the experiment (in order to equate the number of times each of the two objects in each video was the target). In order to make sure that participants understood the task, they ran a 14-trial training block before the beginning of the experiment. The training set used video clips that were equivalent to, but not contained in, the experiment and included examples of the four experimental conditions as well as catch trials.

The experiment contained a total of 168 trials (24 trials per experimental condition plus 72 catch trials; hence, the overall proportion of target-present trials was ∼57%). The experiment was divided into six blocks of 28 videos with a representative number of trials of each condition and catch. Each participant received a different random order of videos.

## Results and Discussion

We ran a repeated measures ANOVA on mean RTs (for correct responses), with subject as the random effect and condition as the factor of interest. The analysis returned a significant main effect of condition [*F*(3,93) = 3.14; *p* = 0.0289]. Given this significant main effect, we went on to test our specific *a priori* predictions using *t*-tests. In particular we had hypothesized that target-consistent characteristic sounds will help attract attention to the corresponding visual object. Based on this hypothesis, we predicted that reaction times should be faster in the target-consistent condition than in the distractor-consistent, neutral, and no sound conditions. The analysis demonstrated that responses in the target-consistent condition were faster than in distracter-consistent [*t*(31) = 2.36, *p* = 0.012, Cohen’s *d* = 0.27], neutral [*t*(31) = 2.33, *p* = 0.013, Cohen’s *d* = 0.39], and no sound [*t*(31) = 2.53, *p* = 0.008, Cohen’s *d* = 0.32] conditions. All these comparisons are one tail (given the directional hypothesis) and survived the multiple comparison correction using Holm–Bonferroni (Ludbrook, 1998).

The second prediction stated that if audio–visual semantic congruence attracts attention in natural scenes automatically even when the objects are irrelevant to the current behavioral goal, then one should expect a slowdown in responses to targets in distracter-consistent trials, with respect to neutral sound and no sound condition. *Post hoc t*-test showed the lack of difference between distractor-consistent and neutral conditions *t*(31) = 0.28, *p* = 0.39. For completion, we also performed non-planned *t*-tests (two-tails) between distractor-consistent and no sound *t*(31) = 0.28, *p* = 0.39, and between neutral and no sound conditions *t*(31) = 0.33, *p* = 0.37. Neither of these comparisons resulted significant. The latter comparison suggests that no cross-modal effect was observed in this experiment due to unspecific general alerting influence of sounds.

To ensure that there was no speed–accuracy trade-off we analyzed error data. The analysis showed that there was no difference in performance between conditions (Figure 3B). Since catch trials do not contain target and distractor objects, the false alarm rate was calculated between three conditions: consistent (when sound corresponds to the search cue word), inconsistent (when the sound does not correspond to the search cue word), and no sound (Figure 3C). The analysis showed no difference in consistent vs. inconsistent trials [*t*(31) = 1.37, *p* = 0.09] and consistent vs. no sound [*t*(31) = 0.44, *p* = 0.33]. However, in inconsistent trials participants had higher false alarm rate in comparison to the no sound condition [*t*(31) = 2.74, *p* = 0.005]. Analysis of miss rates showed no difference between conditions (Figure 3D). The increase in false alarms for catch trials in the inconsistent condition is surprising, because it would mean that participants tend to respond more when the cue word and the characteristic sound are different, rather than the same. Recall that in these trials, there are no visual objects that correspond to either. If this result was to reflect an actual response bias toward being more liberal in inconsistent trials (hence, make more false detections and/or responding faster), this bias would be against the main result detected in the experimental trials.

Over all, the results to emerge from the present study show that, when searching for objects in real-life scenes, target-consistent sounds speed up search latencies in comparison to neutral sounds or when only background noises are present. Instead, distracter-consistent sounds produced no measurable advantage or disadvantage with respect to these baseline conditions (albeit, responses were slower than for target-consistent conditions). This finding demonstrates, for the first time, that characteristic sounds improve visual search not only in simple artificial displays (Iordanescu et al., 2008, 2010) but also in complex dynamic visual scenes with contextual information. In general, and according to previous studies (Iordanescu et al., 2008, 2010), we can affirm that the results obtained in this study are due to object-based and not due to spatiotemporal correspondences since we avoided any kind of spatiotemporal congruence. Semantic relationships between the objects in a complex visual scene can guide attention effectively (Wu et al., 2014, for review), our results suggest that this semantic information did not make congruent auditory information redundant. Semantically consistent sounds can indeed benefit visual search along with available visual semantic information. This is the novel contribution of this study.

Despite research on attention orienting has been dominated primarily by low-level spatial and temporal factors (salience), recent research has focused on the role of higher-level, semantic aspects (e.g., Henderson and Hayes, 2017). Visual-only studies have highlighted, for example, the importance of functional relationships between objects (Biederman et al., 1982; Oliva and Torralba, 2007; MacEvoy and Epstein, 2011), expectancies regarding frequent spatial relations (Peelen and Kastner, 2014, for review), and cues to interpersonal interactions (Kingstone et al., 2003; Papeo et al., 2017, 2019) as important in determining some aspects of visual scene perception. These factors are to play an especially important role in real-life naturalistic scenarios, where these high-level relationships are often abundant (Peelen and Kastner, 2014). Adding to this evidence from visual-only experiments, in the present study we demonstrated that high-level cross-modal (auditory–visual) semantic relations may as well exert an impact in spatial orienting and guide attention in visual search for objects in real-life, dynamic scenes. In fact, one could speculate that especially in complex and noisy environments where many visual and auditory events are spatially and temporally coincident, semantic information might become a leading predictor of object presence, and hence, guide attention.

The visual and auditory materials we used in our study are highly heterogeneous; therefore, it is very challenging to control for all the possible compounds such as movement, presence of people in videos, size, and position of objects, physical salience, and meaning of the scene. We addressed these differences between videos by counterbalancing them across subjects. However, this does not allow us to completely discard the possible influence of the stimulus properties on orienting behavior and therefore on the results of the study. Another possible issue might be the absence of distinction in our study between sounds that either physically or semantically are close to each other, e.g., sound of a guitar and sound of the piano (the same semantic group of musical instruments) or the sound of the coins or keys (physically similar). This way we cannot be sure that sound from the same semantic category or sound that is physically similar could play a proper role of a distractor or neutral sound.

In the current study, we used a detection task (pressing the button as soon as the target object is found). One may argue that this design does not allow us to assure that participants respond to the target and not for the distractor. Since the videos are very heterogeneous, it was not possible to design discrimination instead of a detection task while preserving control of the relevant variables. Catch trials were introduced in the experiment specifically to avoid (and control) excessively liberal response criteria (high proportion of “yes” guessing responses). However, we did not anticipate any particular hypothesis regarding false alarms in different conditions and because of this catch trials did not contain sound-congruent distractor objects. This way our design does not allow to calculate false alarm rate for the distractor-consistent trials. One possible concern which could be raised is that participants were responding to the sound rather than cue-word, which would still generate correct responses in the target-consistent and target-inconsistent trials. However, if this happened, we should observe a difference in reaction time data between all target-present trials (consistent and inconsistent) and the neutral sound condition. In particular, since in neutral trials the presented sound does not correspond to any object in the scene, it will probably take more time for participants to respond since they will be looking for something that is not there. This effect is not present in the data of the current study.

Another possible limitation of our design is that distractors that are consistent with the characteristic sound could have induced responses. These responses would compete with the actual correct detection in the target-inconsistent sound condition but could be counted as correct in the target-consistent conditions, hence generating the observed difference between these two conditions in our data. How can we address this possible limitation? If this effect of cue-sound competition had a sizable effect on response patterns, then reaction times and accuracy should decay in the target-inconsistent condition in comparison to the neutral condition (in which no visual objects coincided with the distractor sound and competed for response). However, no differences in reaction times or accuracy between distractor-consistent and neutral conditions were found (we elaborate on this point in the next paragraph). False-alarm data could be potentially informative in this case but unfortunately the design of this study does not allow us to calculate false alarm for distractor-consistent condition (see above). One prior study by Knoeferle et al. (2016) measured false alarm rates in a similar visual search task with simpler scenes and the same conditions for characteristic sounds. Knoeferle et al. (2016) reported no differences in false alarms between conditions in five experiments with an exception of marginal tendency for distractor-congruent sound compared to the no-sound condition in two of the experiments. Therefore, based on that study it seems that incongruent sound does not strongly bias participant to confuse target with the distractor. However, we must be careful in extrapolating these assumptions to the current data.

Another open question is why target-consistent sounds benefit search, but distracter-consistent sounds do not slow down reaction times (in comparison to neutral or no sounds). If cross-modal interactions were strictly automatic and pre-attentive, then distractor sounds should increase the saliency of their corresponding, yet irrelevant objects present in the scene. However, the evidence we found is not consistent with the strong pre-attentive view of cross-modal semantic effects. Despite the interplay between attention and multisensory interactions is far from resolved (Talsma et al., 2010; Ten Oever et al., 2016; Hartcher-O’Brien et al., 2017; Lunn et al., 2019; Soto-Faraco et al., 2019, for some reviews), many studies illustrate that multisensory interactions tend to wane when the implicated inputs are not attended (e.g., Alsius et al., 2005, 2014; Talsma and Woldorff, 2005). For example, Molholm et al. (2004) demonstrated that object-based enhancement occurs in a goal-directed manner, suggesting that while a characteristic sound of a target will facilitate its localization, a characteristic sound of a distracter will not attract attention to the distracter. In line with Molholm et al. (2004) and other previous studies (Iordanescu et al., 2008, 2010; Knoeferle et al., 2016) in our study we demonstrated that in visual search task semantically consistent sound helps to find a visual target faster. This might be due to the fact that auditory encoding of a sound, e.g., a barking sound enhances visual processing of all the features that are related to a dog. This way all the auditory and visual semantic associations are likely to develop simply because of repeated coincidence when experiencing the multisensory object. At first, the cue word activates the semantic web of the target of search and creates an attentional template for the search. Further, the characteristic sound reinforces this activation and therefore the object is found faster. However, it remains unknown if the semantically congruent audio–visual event can attract attention in an automatic way when it is not relevant to the task or when there is no task at all (e.g., free observation).

Consistent with the idea of automaticity [and therefore, contrary Molholm et al. (2004) and to our results], a study by Mastroberardino et al. (2015) showed that audio–visual events can capture attention even when not task-relevant. Here, it is important to note that our design was not necessarily optimized to detect such distractor-consistent effect (e.g., as discussed above, it was not sensitive enough in terms of detecting distractor-induced false alarms). There are other important differences between the present study and Mastroberardino et al. (2015), which could account for the fact that task-irrelevant semantic audio–visual congruency could have had a larger impact. For example, Mastroberardino et al. (2015) used a low perceptual load situation with a very limited range of possible semantic relationships (just two). We believe that object-based cross-modal enhancements might eventually occur even when task-irrelevant, under favorable low load conditions. Further studies to understand the limits of cross-modal semantic effects and how they apply to real-life dynamic scenarios should be run to clarify this point. For example, in line with the present study, a possible next step would be to use eye-tracking with free-viewing of the video-clips to investigate if cross-modal semantic congruency attracts visual behavior and can be, therefore, responsible for the visual search effects seen here.

## Conclusion

In conclusion, we have demonstrated that semantic consistent sounds can produce an enhancement in visual search in complex and dynamic scenes. We suggest that this enhancement happens through object-based interactions between visual and auditory modalities. This demonstration not only generalizes (and confirms) previous laboratory findings on semantically based cross-modal interactions but also expands it to the field of research in natural scenes.

## Data Availability Statement

The datasets generated for this study are available on request to the corresponding author.

## Ethics Statement

The studies involving human participants were reviewed and approved by the Clinical Research Ethics Committee (CEIC) of Parc de Salut Mar UPF. The patients/participants provided their written informed consent to participate in this study.

## Author Contributions

DK, LG-V, and SS-F contributed to the conception and design of the study. DK and LG-V prepared the stimuli and collected the data. DK and LG-V performed the statistical analysis. DK wrote the manuscript. All authors contributed to the manuscript revision, and read and approved the submitted version of the manuscript.

## Conflict of Interest

The authors declare that the research was conducted in the absence of any commercial or financial relationships that could be construed as a potential conflict of interest.

The reviewer, EM, declared a past collaboration, with one of the authors, SS-F, to the handling Editor.

## Footnotes

1 https://www.techsmith.com/camtasia/

2 www.blackboxtoolkit.com
